# Association of ultrasonographically determined anatomical location of Bethesda 3 thyroid nodules with differentiated thyroid cancer

**DOI:** 10.5339/qmj.2022.51

**Published:** 2022-11-14

**Authors:** Emre Gezer, Halil Demirkan, Zeynep Cantürk, Alev Selek, Berrin Çetinarslan, Mehmet Sözen, Mustafa Çalişkan, Alper Çağrı Karci, Tevhide Bilgen Özcan, Ömercan Topaloğlu, Büşra Yaprak Bayrak, Özge Demirkol

**Affiliations:** ^1^Faculty of Medicine, Kocaeli University, Department of Endocrinology and Metabolism, Kocaeli, Turkey Email & ORCID ID: gezer_emre@hotmail.com & https://orcid.org/0000-0002-5340-6106; ^2^Adatip Hospitals, Division of Endocrinology and Metabolism, Sakarya, Turkey; ^3^Duzce Ataturk State Hospital, Division of Endocrinology and Metabolism, Duzce, Turkey; ^4^Bağcılar Training and Research Hospital, Department of Endocrinology and Metabolism, Istanbul, Turkey; ^5^Bağcılar Training and Research Hospital, Department of Pathology, Istanbul, Turkey; ^6^Zonguldak Bulent Ecevit University, Department of Endocrinology and Metabolism, Zonguldak, Turkey; ^7^Faculty of Medicine, Kocaeli University, Department of Pathology, Kocaeli, Turkey

**Keywords:** Aspiration biopsy, fine-needle, cytopathology, undetermined significance, thyroid nodule, ultrasonographic imaging, location, papillary thyroid carcinoma

## Abstract

Background: The size of a thyroid nodule and certain sonographic features, such as marked hypoechogenicity, microcalcifications, taller-than-wide shape, microlobulated, or irregular margins, indicate the greater malignancy risk. The frequency of the atypia of undetermined significance/follicular lesion of undetermined significance (AUS/FLUS) category among cytology reports from thyroid fine-needle aspirations ranges from 0.8% to 28%, whereas the risk of malignancy of these nodules varies from 6% to 97%. This retrospective analysis investigated whether the preoperative ultrasonographic location of Bethesda 3 thyroid nodules is a predictive risk factor for differentiated thyroid cancer (DTC).

Methods: A total of 387 patients who underwent total thyroidectomy for a nodule with AUS/FLUS cytology and diagnosed with a DTC at five tertiary referral centers between 2010 and 2020 were retrospectively analyzed. The location of the thyroid nodule with AUS/FLUS cytology was categorized into two groups: one group was composed of the isthmus, upper lobe, middle lobe, and lower lobe, whereas the latter consisted of right lobe, left lobe, and isthmus.

Results: DTC was diagnosed in 40.6% (n = 157) of the operated nodules. Multiple logistic regression analysis has revealed that hypoechogenicity of the nodule (odds ratio [OR] = 2.929, *p* < 0.001) was the only independent predictive factor for the malignancy of the nodules with AUS/FLUS, whereas the location of the nodule, age, and sex were not significantly independent risk factors. Multifocality and contralateral benign nodules were independent predictive factors for multicentricity (OR = 3.5, *p* = 0.002; OR = 5.5, *p* = 0.001, respectively).

Conclusion: As the first study investigating the association between a Bethesda 3 nodule location and the risk of malignancy by evaluating postoperative cytology reports, the results showed that nodule location with AUS/FLUS on fine-needle aspiration biopsy was not a predictive risk factor for the diagnosis of DTC.

## Introduction

Differentiated thyroid cancers (DTC) include papillary and follicular cancers. According to the Surveillance, Epidemiology, and End Results database from 1975 to 2018, the incidence of papillary cancer is approximately 13.5 per 100,000 by 2018.^1^ Through the routine use of ultrasonography (US) in clinical practice, the rate of thyroid nodules in the general population has increased up to 65%. The thyroid nodules are mainly evaluated by US to determine the cancer risk stratification, and in some clinics, including the department of radiology, US is performed according to the European Thyroid Imaging and Reporting Data System (EU-TIRADS).^2^ Like many other systems, this system evaluates nodule size and certain sonographic features, such as marked hypoechogenicity, microcalcifications, taller-than-wide shape, microlobulated, or irregular margins, which indicate greater malignancy risk (TIRADS 5); however, the relationship between the location of the nodules and the risk of malignancy had not been reported prior to a poster presented in 2018 by Zhang *et al.* ^3^

Fine-needle aspiration biopsy (FNAB) is the gold standard method to identify the cytologic features of the nodules for the clinical evaluation of malignancy. According to the Bethesda System for Reporting Thyroid Cytopathology, six categories have been described: nondiagnostic/unsatisfactory, benign, atypia of undetermined significance/follicular lesion of undetermined significance (AUS/FLUS), follicular neoplasm/suspicious for a follicular neoplasm, suspicious for malignancy, and malignant.^4^ In several large studies, the frequency of the AUS/FLUS category among cytology reports from thyroid fine-needle aspirations has ranged from 0.8% to 28%, whereas the risk of malignancy of these nodules varies between 6% and 97%.^5–7^

To determine if the location of a thyroid nodule is an additional risk factor for malignancy to prevent overtreatment with unnecessary surgeries, several studies since 2018 have investigated this relationship,^8–11^ in which all studies have evaluated data from FNAB, but no evaluation was conducted for postoperative pathology reports. Two large studies have shown the significantly higher risk of malignancy in isthmic nodules compared with other locations in the gland.^8,9^ This significant association has also been demonstrated for nodules located in the upper and middle poles of the thyroid gland in two other studies.^3,11^ By contrast, a recent study reported that the isthmic location of thyroid nodules was not related to any Bethesda System category.^10^ Regarding these outcomes in this novel research area, a retrospective analysis was conducted to investigate whether the preoperative ultrasonographic location of Bethesda 3 thyroid nodules is a predictive risk factor for DTCs by evaluating postoperative pathology reports.

## Methods

All data of patients who underwent total thyroidectomy for a nodule with AUS/FLUS cytology, indicated by FNAB, and diagnosed with a DTC (papillary or follicular thyroid cancer, including noninvasive follicular thyroid neoplasm with papillary-like nuclear features) or a benign adenoma at five tertiary referral centers, which approved to participate in this study by sharing their databases within the northwest region of Turkey between 2010 and 2020, were retrospectively analyzed and included in the study. The exclusion criteria were as follows: concomitant diagnosis of medullary or anaplastic cancer; ≥ 4 cm nodule; any ≥ Bethesda category 3 nodules in the thyroid gland before the operation; Bethesda 3 nodule occupying the entire thyroid lobe; extending nodule with AUS/FLUS cytology between two different regions of the thyroid lobe; presence of TIRADS 5 nodules, nodules with extrathyroidal extension; non-specialist radiological or pathological reports; history of radiotherapy to the head, neck, or chest wall; history of multiple endocrine neoplasia 2; family history of any thyroid carcinoma; age <  18 years; and missing data (exact location or features of Bethesda 3 nodules on US).

After excluding numerous patients, mainly due to the missing data of nodule location or features on US, a total of 387 patients from five tertiary centers were included in this study. Each thyroid nodule was described in terms of size, echogenicity, margin status, and internal composition by US and reported according to EU-TIRADS. US was performed by specialist radiologists, and cytological evaluation of FNABs and postsurgical resected specimens were reported by specialist pathologists in each of five participating centers.

The location of thyroid nodules with AUS/FLUS cytology was categorized into two groups: one group was composed of the isthmus, upper lobe, middle lobe, and lower lobe, whereas the other consisted of the right lobe, left lobe, and isthmus. In addition to the exact location of the nodule, preoperative and postoperative data of the patients, such as sex, age at diagnosis, ultrasonographic features of the Bethesda 3 nodule (hypoechogenicity and maximum diameter), presence of any other nodule in the ipsilateral or contralateral lobe preoperatively, pathological features of the resected thyroid specimens (size, multifocality, and multicentricity of the tumor), were noted. Multifocality was defined as > 1 malignant focus in the ipsilateral lobe of the primary tumor and multicentricity as the presence of any malignant tumor in the contralateral lobe.

All statistical analyses were performed using the IBM SPSS Statistics for Windows version 20.0 (IBM Corp., Armonk, NY, USA) and R 3.6.2. The Kolmogorov–Smirnov test was used to assess the assumption of normality. Continuous variables were presented with mean ±  standard deviation or median (25^th^–75^th^ percentile). Categorical variables were summarized as counts (percentages). Between-group comparisons of continuous variables were conducted using the independent samples t-test for normally distributed data and the Mann–Whitney U test for non-normally distributed data. The association between two categorical variables was examined by the Chi-square test. Predictors of malignancy were identified with multiple logistic regression using nodule location as the dependent variable. In all statistical analyses, a two-sided *p* value of <  0.05 at 95% confidence level was considered significant.

The study was approved by the ethics committee of Kocaeli University, Faculty of Medicine (October 26, 2020, No. KÜ GOKAEK 2020/18.28 - 2020/317).

## Results

This study included a total of 387 patients who underwent total thyroidectomy or lobectomy for preoperatively detected unilateral Bethesda 3 nodules. The majority (86.3%) of the patients were women. DTC was diagnosed in 40.6% of the operated nodules. Table 1 presents the demographic and clinical characteristics of the patients including the location of unilateral Bethesda 3 nodules on US and multifocality/multicentricity status of malign nodules.

On the univariate analysis, nodule localization within the lobe, nodule lateralization, sex, age, existence of other nodules preoperatively in the ipsilateral and contralateral lobes, and maximum diameter of the nodule were not significantly correlated with the malignancy risk of the operated nodules. Hypoechogenicity was the only variable that was significantly associated with the malignancy risk of Bethesda 3 nodules (Table 2).

In line with the univariate analysis, multiple logistic regression analysis also revealed that hypoechogenicity of the nodule (odds ratio [OR] = 2.929, *p* = < 0.001) was the only independent predictive factor for the malignancy of nodules with AUS/FLUS, whereas nodule location, age, and sex were not significantly independent risk factors (Table 3). Similarly, multiple logistic regression analysis of nodule lateralization along with other adjusted factors showed the lack of association between the thyroid cancer diagnosis and nodule location (right lobe vs isthmus, OR = 0.950, *p* = 0.917; left lobe vs isthmus, OR = 0.813, *p* = 676) (Table 3). In addition to hypoechogenicity, age and sex were included in this analysis even if these variables were found as not significantly associated factors in the univariate analyses. These two variables were included to allow for the calculation of an adjusted OR of the correlation between the location of the nodule with AUS/FLUS and malignancy, after excluding the significant effects of age and sex on malignancy risk, as described in the literature.

In the univariate analysis of 157 patients with DTC, a strong significant association was found between multicentricity and multifocality on pathology and contralateral benign nodule on US (*p* = 0.002 and *p* < 0.001, respectively), and other factors such as age, sex, hypoechogenicity, another nodule in the ipsilateral lobe, and nodule size on US did not show any significant relationship with multicentricity (Table 4). Similarly, the adjusted model that included multifocality, contralateral benign nodule, and location of Bethesda 3 nodule showed that the first two parameters were independent predictive factors for multicentricity (OR = 3.5, *p* = 0.002; OR = 5.5, *p* = 0.001, respectively). The location of the nodule with AUS/FLUS was not associated with multicentricity (Table 3).

## Discussion

In this study, an analysis of 387 patients who underwent total thyroidectomy or lobectomy for preoperatively detected unilateral nodule with AUS/FLUS, Bethesda 3 thyroid nodule location was shown to be not a risk factor for DTC diagnosis even after adjusting for other significant predictors of thyroid cancer such as hypoechogenicity, sex, and age. To date, only five studies have examined the association of thyroid cancer with the location of a thyroid nodule, and all these studies have evaluated the data retrieved from FNAB reports.^8–11^ In a recent study from Brazil, the location of the 3701 thyroid nodules was divided into three, i.e., isthmus, right lobe, and left lobe, and no significant correlation was found between Bethesda categories and nodule location.^10^ In accordance with this study, the results have also revealed the lack of a significant difference in the DTC risk of nodules with AUS/FLUS, between the isthmus, right lobe, and left lobe.

The first study that examined the malignancy predictivity of nodule location was conducted by Zhang *et al.*, and the multiple logistic regression model demonstrated that the odds of malignancy in upper pole nodules were significantly higher than that of nodules in other poles.^3^ Proposed mechanisms for that elevated risk include the higher doses of X-ray radiation in the upper poles during dental examinations or computed tomography of the head^12^ and the accumulation of reactive oxygen species in the upper lobe, which induces the cancer-promoting mutations caused by the circuitous venous drainage route of the upper lobes.^13^ In 2018, Ramundo *et al.* evaluated FNAB cytology reports of 227 thyroid nodules and reported that the odds for malignancy were significantly higher for middle lobe nodules than for lower pole nodules, even after adjusting for high-risk estimation according to the EU-TIRADS US classification system, while no significant elevated risk was found for upper pole nodules.^11^

The largest study was conducted by Pastorello *et al.*, who evaluated 9535 cytology reports from FNAB and postoperative histopathological reports of 915 patients who underwent a surgical resection. That study reported the percentage of malignancy of isthmic nodules was significantly higher than that of the right and left lobe nodules.^8^ A study from the United States, in which 3313 patients with 3419 thyroid nodules were included, showed that isthmus nodules had the highest risk of malignancy compared with upper, lower, and middle lobe nodules based on FNAB reports in a multivariate regression model.^9^ This preponderance might be related to the smaller percentage of nondiagnostic samples among isthmic nodules because of the more superficial anatomical location of isthmic nodules compared with nodules located in other parts of the thyroid.^8^ Another possible mechanism has been attributed to the embryological development of the gland. Isthmic cells could have a different composition from other cells in the other two lobes. During the embryologic period, the two lateral lobes developed from the ultimobranchial bodies, whereas the isthmus developed from the primitive pharynx.

In contrast to all these studies that evaluated FNAB data of thyroid nodules, this is the first study to investigate the cancer risk predictivity of Bethesda 3 thyroid nodule location by evaluating the postoperative histopathological reports, and the results indicated that location, i.e., upper, middle, and lower poles, isthmus, and right and left lobes, was not a predictive risk factor for DTC. In agreement with various reports, hypoechogenicity was found to be an independent risk factor associated with the malignancy of nodules with AUS/FLUS.^14^

In this study, the incidence rate of contralateral carcinoma was 28.0% (44 of 157), which is higher than that previously reported in a few articles,^15^ while it is similar to that in a study that examined 243 patients with papillary thyroid carcinoma.^16^ In the literature, multiple preoperative US and postoperative histopathologic features have been reported as predictive for an occult contralateral thyroid carcinoma.^15,17–21^ The majority of these studies has agreed on the multifocality in the ipsilateral lobe on pathology as a common predictive risk factor,^17–19,21^ whereas the other common risk factor has been reported to harbor a contralateral benign nodule.^15,18,21^ Moreover, some data about contralateral cancer predictivity of BRAF mutation, age, and tumor size tumor are controversial.^15,17,19,20^ In agreement with the results of most studies, in the present study, the analysis of 157 patients with DTC showed that multifocality on pathology and contralateral benign nodule on US were independent predictive risk factors for multicentricity, whereas other factors such as age, sex, hypoechogenicity, another nodule in the ipsilateral lobe, and nodule size on US did not show a significant relationship with multicentricity.

Owing to the comprehensive exclusion criteria in this study, especially the missing data on the exact location of Bethesda 3 nodule and the retrospective nature of the study, numerous patients were excluded, which was the main limitation of this study. In addition, only one preoperative US report of each patient had been evaluated, and patient data had been collected from five different centers. A prospective study in a single center in which a single radiologist evaluates the patients could discard the inconsistent US reports with missing data from various imaging centers.

## Conclusion

The location of nodules with AUS/FLUS on FNAB was not a predictive risk factor for the diagnosis of DTC. To the best of our knowledge, this is the first study to investigate the association between ultrasonographic location of a Bethesda 3 nodule and malignancy risk by evaluating postoperative cytology reports. As a result, the decision on surgical approach toward Bethesda 3 nodules may be given according to the well-known criteria previously described in the guidelines that recently focus on the significance of molecular studies, regardless of the nodule location.

## Figures and Tables

**Table 1:** Demographic and clinical characteristics of the patients (n = 387)

Age (years), mean ± SD	48.0 ± 11.8n (%)

Sex	

Female	334 (86.3)

Male	53 (13.7)

Locations of the nodules	

Superior	45 (11.6)

Middle	124 (32.0)

Inferior	197 (50.9)

Isthmus	21 (5.4)

Lateralization of the nodules	

Right lobe	194 (50.1)

Left lobe	172 (44.4)

Isthmus	21 (5.4)

Hypoechogenicity	

Yes	176 (45.5)

No	211 (54.5)

Other nodules in the ipsilateral lobe	

Yes	131 (33.9)

No	109 (28.2)

Other nodules in the contralateral lobe	

Yes	162 (41.9)

No	78 (20.2)

Postoperative histopathology	

Malign	157 (40.6)

Benign	230 (59.4)

Multifocality	

Yes	48 (30.6)

No	109 (69.4)

Multicentricity	

Yes	44 (28.0)

No	113 (72.0)


**Table 2:** Association of the demographic data of the patients and ultrasonographic features of the nodule with the malignancy risk of a 0Bethesda 3 nodule (n = 387)

	Benign (n = 230)	Malign (n = 157)	p^^*^^

Age (years) mean ± SD	48.8 ± 11.4	46.9 ± 12.3	0.122

Location n (%)			

Superior	25 (55.6)	20 (44.4)	0.827

Middle	71 (57.3)	53 (42.7)	

Inferior	121 (61.4)	76 (38.6)	

Isthmus	13 (61.9)	8 (38.1)	

Lateralization			

Right	111 (57.2)	83 (42.8)	0.660

Left	106 (61.6)	66 (38.4)	

Isthmus	13 (61.9)	8 (38.1)	

Sex			

Female	195 (58.4)	139 (41.6)	0.336

Male	35 (66.0)	18 (34.0)	

Hypoechogenicity			

Yes	80 (45.5)	96 (54.5)	< 0.001

No	150 (71.1)	61 (28.9)	

Existence of other nodules in the ipsilateral lobe			

Yes	47 (35.9)	84 (64.1)	0.644

No	36 (33.0)	73 (67.0)	

Existence of other nodules in the contralateral lobe			

Yes	62 (38.3)	100 (61.7)	0.083

No	21 (26.9)	57 (73.1)	

	Median (25^th^–75^th^)	Median (25^th^–75^th^)	p^**^

Max diameter of the nodule on US (mm)	19.00 (13.00–25.00)	18.00 (13.00–23.75)	0.312


^*^Evaluated by the Chi-square test

^**^Evaluated by the Mann–Whitney U test

**Table 3:** Multiple logistic regression analyses of the adjusted factors, correlation with malignancy and multicentricity

Correlation with malignancy			

Group 1	OR	95% CI for OR	p

Hypoechogenicity	2.929	1.911-4.489	< 0.001

Sex-M vs F	0.742	0.391-1.408	0.361

Age	0.992	0.974-1.010	0.390

Location of the nodule			

Middle vs lower	1.196	0.744-1.925	0.460

Upper vs lower	1.269	0.640-2.515	0.496

Isthmus vs lower	1.239	0.471-3.262	0.664

Group 2			

Hypoechogenicity	2.921	1.907-4.474	< 0.001

Sex-M vs F	0.739	0.390-1.400	0.354

Age	0.992	0.974-1.011	0.406

Location of the nodule			

Right lobe vs isthmus	0.950	0.362-2.494	0.917

Left lobe vs isthmus	0.813	0.307-2.152	0.676

Correlation with malignancy			

Contralateral benign nodule	5.525	2.086-14.631	0.001

Multifocality	3.466	1.580-7.602	0.002

Location of the nodule			

Middle vs lower	0.642	0.274-1.506	0.308

Upper vs lower	0.487	0.133-1.780	0.277

Isthmus vs lower	0.594	0.096-3.670	0.575


CI, confidence interval; F, female; M, male; OR, odds ratio

**Table 4:** Association of the demographic data of the patients and ultrasonographic and cytological features of the malignant nodule with multicentricity (n = 157)

	Multicentricity		p

	Yes	No	

Age (years) mean ± SD	46.5 ± 10.5	47.0 ± 12.9	0.79**

Sex n (%)			

Female	39 (28.1)	100 (71.9)	1.00*

Male	5 (27.8)	13 (72.2)	

Hypoechogenicity			

Yes	28 (29.2)	68 (70.8)	0.83*

No	16 (26.2)	45 (73.8)	

Location			

Superior	4 (20.0)	16 (80.0)	0.76*

Middle	14 (26.4)	39 (73.6)	

Inferior	24 (31.6)	52 (68.4)	

Isthmus	2 (25.0)	6 (75.0)	

Existence of other nodules in the ipsilateral lobe			

Yes	28 (33.3)	56 (66.7)	0.16*

No	16 (21.9)	57 (78.1)	

Existence of other nodules in the contralateral lobe			

Yes	38 (38.0)	62 (62.0)	< 0.001*

No	6 (10.5)	51 (89.5)	

Multifocality			

Yes	22 (45.8)	26 (54.2)	0.002*

No	22 (20.2)	87 (79.8)	

	Median (25^th^-75^th^)	Median (25^th^-75^th^)	

Max diameter of the nodule on US (mm)	15.00 (12.00-22.00)	18.00 (13.00-24.00)	0.24^a^


^*^Evaluated by the Chi-square test

^**^Evaluated by the independent t-test

^α^Evaluated by the Mann–Whitney U test
